# Receptor-Interacting Protein Kinase-3 Expression Impacts Ocular Vascular Development and Pathological Neovascularization

**DOI:** 10.3390/cells13242109

**Published:** 2024-12-20

**Authors:** Yong-Seok Song, Shoujian Wang, SunYoung Park, Barbara Hanna, Kelsey J. Johnson, Soesiawati R. Darjatmoko, Mohammad Ali Saghiri, Ali Mohammad Saghiri, Bo Liu, Christine M. Sorenson, Nader Sheibani

**Affiliations:** 1 Department of Ophthalmology and Visual Sciences, University of Wisconsin School of Medicine and Public Health, Madison, WI 53705, USA; song224@wisc.edu (Y.-S.S.); shoujianwang@wisc.edu (S.W.); spark67@wisc.edu (S.P.); srdarjat@wisc.edu (S.R.D.); 2 McPherson Eye Research Institute, University of Wisconsin School of Medicine and Public Health, Madison, WI 53705, USA; 3 Department of Pediatrics, University of Wisconsin School of Medicine and Public Health, Madison, WI 53705, USA; hanna.barbara@mayo.edu (B.H.); kjohnson92@wisc.edu (K.J.J.); 4 Department of Restorative Dentistry, Rutgers School of Dental Medicine, Newark, NJ 07103, USA; mohammadali.saghiri@rutgers.edu; 5 Department of Computer Science, William Paterson University, Wayne, NJ 07470, USA; saghiria@wpunj.edu; 6 Department of Cell and Regenerative Biology, University of Wisconsin School of Medicine and Public Health, Madison, WI 53705, USA; bliu24@wisc.edu

**Keywords:** oxygen-induced ischemic retinopathy, choroidal neovascularization, necroptosis, RIPK-proteins, retinal vascularization

## Abstract

Functional cell death pathways are essential for normal ocular vascular development and tissue homeostasis. As our understanding of necrosis-based cell death pathways has expanded, the inclusion of regulated forms, including necroptosis, ferroptosis, and oxytosis, has occurred. Although the existence of these pathways is well described, our understanding of their role during vascular development and pathological neovascularization is very limited. Here, we examined the role of receptor-interacting protein kinase-3 (Ripk3), a key regulator of necroptosis, in postnatal retinal vascularization and retinal and choroidal neovascularization under pathological conditions. Postnatal vascularization of the retinal superficial layer in the absence of Ripk3 (*Ripk3*^−/−^) was not significantly different from wild-type mice. However, we noted decreased retinal endothelial cells and pericyte numbers at 3 weeks of age when the formation of the retinal primary vascular plexus was complete. In contrast, choroidal and retinal neovascularization following laser treatment and oxygen-induced ischemic retinopathy increased in the absence of Ripk3 expression, respectively. In addition, the inhibition of RIPK1/3 activity suppressed choroidal neovascularization. Thus, Ripk3 expression and/or activity may have unique roles during normal and pathological ocular vascularization through its interactions with Caspase 8 and modulation of cell death processes.

## 1. Introduction

Cell death plays an integral role during development and in disease pathogenesis. The removal of cells by death is classically categorized as occurring by apoptosis or necrosis, with both having the potential to be a regulated process. Although apoptosis is well recognized as a regulated form of cell death, it is only recently that necrosis has been seen in the same light with regulated forms, including necroptosis, ferroptosis, and oxytosis. Presently, we have a limited understanding of the role these regulated forms of necrosis play in retinal normal vascularization, neovascularization, and other vascular pathologies.

Necroptosis is a combination of both necrosis and apoptosis. Like necrosis, it results in plasma membrane rupture, but like apoptosis, it requires energy and is a “programmed” process. Necroptosis is a highly proinflammatory process that appears to activate the immune system differently than necrosis [1,2]. It is mediated by receptor-interacting protein kinase-3’s (RIPK3’s) phosphorylation of the mixed lineage kinase-like (MLKL) protein, resulting in plasma membrane permeability and its eventual rupture [3,4]. When Caspase 8 is recruited to the necrosome complex (RIPK1, RIPK3, and MLKL), it mitigates necroptosis by cleaving and inactivating RIPK3. Necroptosis is initiated by multiple types of stimuli, including death receptor and toll-like receptor-activated signaling events instigated by TNF-α, TRAIL, or FasL [5,6] and DAMPS (damage-associated molecular patterns) [7], respectively. In addition, RIPK3 also has necroptosis-independent functions, including the regulation of inflammation, perhaps through its scaffolding interactions, during embryonic development [8,9], metabolism, and oxidative stress [10].

RIPK3 demonstrates roles during development and disease. Its expression can be silenced by methylation in cancer cell lines, potentially allowing tumor cells to avoid treatment regimens, while others have speculated a role for RIPK3 in controlling cancer development and progression [6,11,12]. RIPK3’s expression and activity are also regulated by microRNAs [13,14] and O-GlcNacylation [15], which can impact metabolism [10]. RIPK1, RIPK3, and MLKL are noted in mammals and vertebrates but not in primitive organisms, leading to the supposition that necroptosis facilitates the removal of defective embryos (reviewed in [16]). The aberrant regulation of inflammatory and cell death processes is the mainstay of many disease states. In many pulmonary, hepatic, and kidney diseases that include injury and/or fibrosis, dysregulated RIPK3/necroptosis is proposed to play a contributing role. There are also indications that dysregulated RIPK3 impacts other autoimmune, inflammatory, and neurodegenerative diseases such as Parkinson’s, rheumatoid arthritis, and amyotrophic lateral sclerosis. Thus, a better understanding of the role necroptosis plays during development and in disease states will allow us to design better treatments.

We hypothesized that Ripk3 expression and activity differentially influence normal retinal vascularization as well as pathological retinal and choroidal neovascularization. Here, we examined the role that RIPK3 expression plays during postnatal retinal vascular development and in ocular disease states such as the oxygen-induced ischemic retinopathy (OIR) and laser photocoagulation-induced rupture of the Bruch’s membrane (a model of wet/neovascular age-related macular degeneration; nAMD). Using mice deficient in Ripk3 (*Ripk3*^−/−^), we examined the postnatal progression of developing retinal vasculature. Mouse retinal vascularization starts immediately after birth with the formation of the primary retinal vascular plexus, which is completed by 3 weeks of age. The retinal vasculature continues to undergo remodeling and maturation, which is completed by 6 weeks of age. Using wholemount trypsin digests of retinas prepared from 3-week- and 6-week-old mice, we assessed the density of retinal endothelial cells (ECs) and pericytes (PCs) in wild-type (WT) and *Ripk3***^−/^**^−^ mice. We also assessed the retinal vasculature structure and organization of mature WT and *Ripk3*^−/−^ mice from their fundus images. Utilizing the mouse OIR and laser photocoagulation models, we determined the impact of Ripk3 expression on retinal and choroidal pathological neovascularization, respectively. The impact of Ripk3 expression on angiogenic sprouting was also assessed using aortas prepared from WT and *Ripk3*^−/−^ mice.

## 2. Materials and Methods

### 2.1. Animal Studies

All animal studies were performed in accordance with the Association for Research in Vision and Ophthalmology for utilizing animals in Ophthalmic and Vision Research. Our protocols were approved by the Institutional Animal Care and Use Committee of the University of Wisconsin School of Medicine and Public Health (IACUC Assurance Number D16-00239). Breeder pairs for *Ripk3*^−/−^ mice on the C57BL/6J background (available in the laboratory) [17] and C57BL/6J background (wild-type mice (WT); Jackson Labs, Bar Harbor, ME, USA; Stock#: 000664) were maintained at the University of Wisconsin animal facilities. Rd8 and Rd1 mutations were not observed with screening.

### 2.2. Laser Induced Choroidal Neovascularization (CNV) and OIR

Ketamine hydrochloride (80 mg/kg) and xylazine (10 mg/kg) were used to anesthetize male and female 3-month-old mice. Their pupils were then dilated with one drop of 2.5% phenylephrine hydrochloride and 0.5% tropicamide (Bausch & Lomb, Tampa, FL, USA). Laser photocoagulation (75 µm spot size, 0.1 sec duration, 120 mW) occurred at the 9, 12, and 3 o’clock positions of the posterior pole of each eye using a slit lamp delivery system (OcuLight GL diode laser; Iridex, Mountain View, CA, USA). A handheld coverslip was used as a contact lens to view the retina. Two weeks post laser treatment, enucleated eyes were fixed in 4% paraformaldehyde. Choroid/RPE complex preparations were incubated in blocking buffer (5% fetal calf serum, 20% normal goat serum in PBS) followed by incubation with anti-ICAM-2 (BD Biosciences, San Diago, CA, USA; 553326; 1:500 in 1× PBS with 20% normal goat serum, 20% fetal calf serum, and 0.3% Triton X-100), anti-F4/80 (ThermoFisher Scientific, Carlsbad, CA, USA; 14-4801-82; 1:500), or anti-collagen I (Abcam, Waltham, MA, USA; ab34710, 1:500) and then incubation was performed with the desired secondary antibody (Jackson ImmunoResearch, West Grove, PA, USA; 1:500). The flatmount choroid/RPE complex was viewed by fluorescence microscopy and the images captured in digital format using a Zeiss microscope (Zeiss, Chester, VA, USA). The total area (in µm^2^) of CNV was calculated using ImageJ software version 1.52J (National Institute of Mental Health, Bethesda, MD, USA; http://rsb.info.nih.gov/ij/, accessed on 15 June 2024). In some cases, C57BL/6j male and female 3-month-old mice received an intraperitoneal injection (IP) of GSK’074 (70 µg/250 µL/day): prepared in DMSO, Cremophor EL (Sigma, St. Louis, MO, USA) and H_2_O (1:1:8) or a vehicle (250 µL; DMSO, Cremophor EL and H2O 1:1:8) beginning 2 days before laser photocoagulation and continuing for 7 days after photocoagulation [18]. At the termination of the experiment, the area of neovascularization was assessed as described above.

For OIR, 7-day-old (P7) pups with dams were exposed for 5 days to an atmosphere of 75 ± 0.5% oxygen with the incubator temperature maintained at 23 ± 2 °C. Oxygen was continuously monitored with a PROOX model 110 oxygen controller (BioSpherix Ltd.; Parish, NY, USA). At P17, 5 days after mice were brought back to room air, retinal wholemounts were prepared and stained with anti-collagen IV (Sigma; AB756P, 1:250). Slides were viewed by fluorescence microscopy and images were captured in digital format using a Zeiss microscope (Carl Zeiss, Chester, VA, USA). The central capillary dropout area, in OIR studies, was quantified (percentage of the whole retina area) from the digital images in a masked fashion with Axiovision software (Version 3.1; Carl Zeiss, Chester, VA, USA). We followed the method of Stahl et al. for the quantification of vitreous neovascularization [19].

### 2.3. Trypsin-Digested Retinal Vessel Preparations

Enucleated eyes were fixed with 4% paraformaldehyde for at least 24 h. They were bisected equatorially using a dissecting microscope to remove the retina. The retinas were then washed overnight in distilled water. They were then incubated in 3% trypsin (Electron Microscopy Sciences, Hatfield, PA, USA; 22200), prepared in 0.1 M Tris, 0.1 M maleic acid, at pH 7.8, containing 0.2 M NaF overnight at 37 °C. Retinas underwent four radial cuts for flattening and were mounted on glass slides and then stained with periodic acid-Schiff (PAS) and hematoxylin. PC and EC were distinguished by nuclear morphology, and the numbers on retinal capillaries were determined in a masked fashion. The number of ECs and PCs in four fields of view from the four quadrants of each mid-retina zone (×400) were counted. All methods were performed as we previously described in detail [20].

### 2.4. Visualization of Retinal Vasculature

As previously reported, enucleated eyes were fixed in 4% paraformaldehyde (10 min at room temperature) and then stored in methanol for at least 24 h at −20 °C [21]. Retinas were blocked, incubated with anti-collagen IV (diluted 1:250 in PBS containing 3% bovine serum albumin (Jackson ImmunoResearch; 00-000-162) and 0.3% Triton X-100) with anti-GFAP (ThermoFisher Scientific; 14-9892-82; 1:100 dilution) at 4 °C overnight. Slides were incubated with secondary anti-rabbit antibodies with Alexa Fluor 594 for collagen IV staining (ThermoFisher Scientific; A-11037, 1:400 dilution) and anti-mouse Alexa Fluor 488 for GFAP (ThermoFisher Scientific; A-11001, 1:400 dilution). The vascular progression was calculated as the distance from the optic nerve to the vascularization end point versus the distance from the optic nerve to the termination of the retinal tissue. Tip cell numbers were determined by counting the number of sprouting tips per unit length of the retina.

### 2.5. Aortic Ring Ex Vivo Sprouting Assay

Thoracic aortas of 4-week-old male and female mice were sectioned into 1 mm long aortic rings with eight aortic rings embedded in Matrigel (BD Biosciences; 356235; 0.5 mL of 10 mg/mL) in a 35 mm dish as previously described [22]. Briefly, 35 mm dishes were coated with 0.5 mL of Matrigel and incubated in a 37 °C incubator for 30 min to harden. Aortic ring sections were then placed on Matrigel and allowed to attach, after which DMEM with 1% fetal bovine serum (FBS; 2 mL) was added, and the cultures were fed every other day for 7 days. Photographs were taken with a Nikon microscope and digital camera, which facilitated the determination of the area of sprouting normalized to the aortic ring area using ImageJ software.

### 2.6. Fundus Imaging

WT and *Ripk3*^−/−^ (8 to10-week-old) male and female mice were anesthetized (ketamine 100 mg/kg and xylazine 10 mg/kg) and their pupils were dilated (2.5% phenylephrine and 0.5% tropicamide; Bausch and Lomb Inc.; Bridgewater, NJ, USA). To keep the mice warm during the experiment, a heating pad was used. Both eyes were examined using fundus imaging on a Micron III retinal imaging system (Phoenix Laboratories Inc., Pleasanton, CA, USA) using an equal image depth, the same magnification, and the circular border of the image distinctly in focus. The Angiogenesis Analyzer plug-in for ImageJ to analyze cellular networks [23] was used to analyze fundus images. In some cases, mice were given a single intraperitoneal injection of NaIO_3_ (50 mg/kg prepared fresh in saline; Sigma, S4007). Fundus images were taken, and eyes were harvested 1 week later for RNA preparation, as described below.

### 2.7. RNA Purification and Real-Time qPCR Analysis

Mouse retina or RPE/choroid, and various retinal cells including endothelial cells (RECs), pericytes (RPCs), astrocytes (RACs), and microglia (MG), and choroidal cells including EC (ChEC), ChPC, RPE, and melanocytes (ChMC), were lysed using the Trizol reagent (ThermoFisher Scientific; 15596018). The method of isolation, growth conditions, and characterization of the different cell types from mouse eyes are described in our publications. Total RNA was extracted using an RNeasy mini kit (Qiagen, Maryland, CA, USA; 74014). cDNA synthesis with an RNA to cDNA EcoDry Premix (Takara Bio, San Francisco, CA, USA; 639549) used 1 µg of total RNA. The cDNA was diluted at 1:10 for the qPCR, and samples were run in triplicate for each biological replicate on a QuanStudio3 real-time PCR system (ThermoFisher Scientific) with a TB-green advantage qPCR premix (Takara Bio; 639676). The amplification parameters used the primers for mouse receptor (TNFRSF)-interacting serine-threonine kinase 1 (Ripk1) F: 5′-tgctgggaataaaggcatgt-3′ and R: 5′-aatgatggcctag cctgtgt-3′; mouse receptor-interacting serine-threonine kinase 3 (Ripk3) F: 5′-tgtgggtaaaggagggttcg-3′ and R: 5′-cacgatcttgactgctacatca-3′; mouse mixed lineage kinase domain-like (Mlkl) F: 5′-atcccatttgaaggctgtga-3′ and R: 5′-aacaactcagggcaatcctg-3′; mouse Caspase 8 (Casp8) F: 5′-aaatggcggaactgtgtgac-3′ and R: 5′-tccccgaggtttgttcttca-3′; and mouse ribosomal protein L13a (Rpl13a) F: 5′-tctcaaggttgttcggctgaa-3′ and R: 5′-gccagacgccccaggta-3′ with standard curves generated from known quantities of each target gene from linearized plasmid, as we previously described [24]. The linear regression line for DNA (ng) was determined with relative fluorescent units (RFU) at a threshold fluorescence value (Ct). Gene targets were quantified from tissue extracts comparing RFU at the Ct to the standard curve. This was normalized by the simultaneous amplification of the housekeeping RpL13a.

### 2.8. Data Analysis

We evaluated statistical differences between groups of more than two with one-way ANOVA followed by Tukey’s Multiple Comparison Test using GraphPad Prism 8.0 (GraphPad Software, San Diego, CA, USA). Tukey’s Multiple Comparison Test was performed to determine the significant differences between the means of every possible two groups in all experimental groups. Statistical analysis between two groups was evaluated with Student’s unpaired *t*-test (two-tailed). The mean ± standard deviation is shown. *p* < 0.05 was considered significant. The mean, SD, and *p*-values in all figures are summarized in Table S1.

## 3. Results

### 3.1. Spreading of the Retinal Vascular Superficial Layer in the Absence of Ripk3

**Figure 1:**
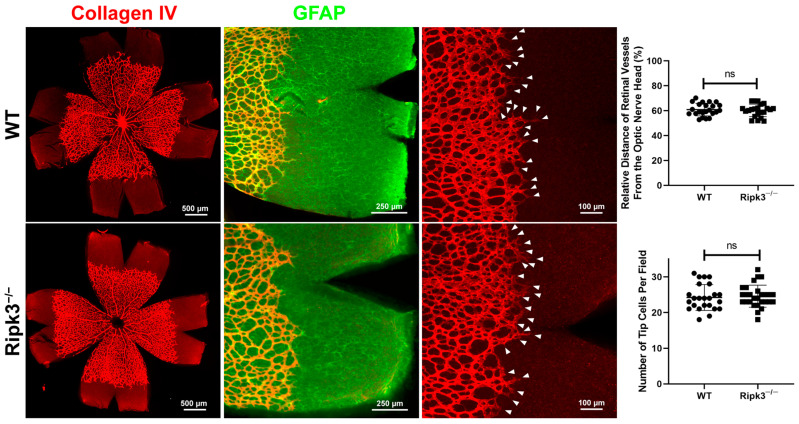
*Ripk3*^−/−^ mice demonstrate a similar rate of spreading as WT mice in the superficial layer. Retinas from P5 male and female mice were wholemount, stained with anti-collagen IV and anti-GFAP, and the distance the retinal vessels spread relative to the radius of the retina was assessed in each quadrant (*n* > 22; ns: not significant). The number of tip cells was also determined, as detailed in the methods. Please note that the spreading of retinal vessels, astrocytes, and tip cell numbers in *Ripk3*^−/−^ mice were not significantly different from WT mice. Student’s unpaired *t*-test (two-tailed).

The proper regulation of cell death is important during development. Unfortunately, we have only a modest understanding of the role necroptosis plays during vascular development. Here, we assessed the influence of Ripk3 expression on the progression of the developing retinal superficial vascular layer (Figure 1). In mice, retinal vascularization occurs postnatally, first forming a superficial layer that is followed by the deep and intermediate layers. The retinal vascular superficial layer begins to form at birth near the optic nerve, reaching the periphery around postnatal day 7 (P7). Here, retinas from P5 (when retinal vasculature is spread near half the distance from the retinal periphery) in WT and *Ripk3*^−/−^ mice were stained with anti-collagen IV (vascular marker; red) and anti-GFAP (glial fibrillary acidic protein; astrocyte marker, green) to assess superficial vascular layer progression and spreading of the retinal astrocytes ahead of the formation of vasculature. Figure 1 demonstrates a similar rate of vascularization of the superficial vascular plexus in retinas from *Ripk3*^−/−^ mice compared to WT mice. We also noted the similar spreading of astrocytes ahead of the developing vasculature (Figure 1). Furthermore, the mean number of sprouting tips per unit retina length was not significantly different in *Ripk3*^−/−^ mice compared with WT mice.

### 3.2. Decreased PC and EC Numbers in Retinas from Ripk3^−/−^ Mice

Once the primary retinal vascular plexus is formed by 3 weeks of age (P21), it is fine-tuned to align with the oxygen requirements of the tissue. As a result, unnecessary retinal vessels are eliminated by a pruning and remodeling process that concludes by 6 weeks of age (P42). Here, EC and PC numbers per unit of the retinal area were counted from retinal trypsin digests of 3- and 6-week-old mice (Table 1). Retinal EC and PC numbers from 3-week-old *Ripk3*^−/−^ mice were significantly lower than their WT counterpart (Table 1). At 6 weeks of age, retinal EC and PC numbers in WT and *Ripk3*^−/−^ mice were comparable. As a result of remodeling and maturation, WT mice displayed decreased EC and PC numbers at 6 weeks compared to 3 weeks of age; consistent with our previous studies, EC and PC numbers were similar in *Ripk3*^−/−^ mice at both 3 and 6 weeks. Thus, Ripk3 expression influences retinal EC and PC numbers during postnatal retinal vascular development.

**Figure 2:**
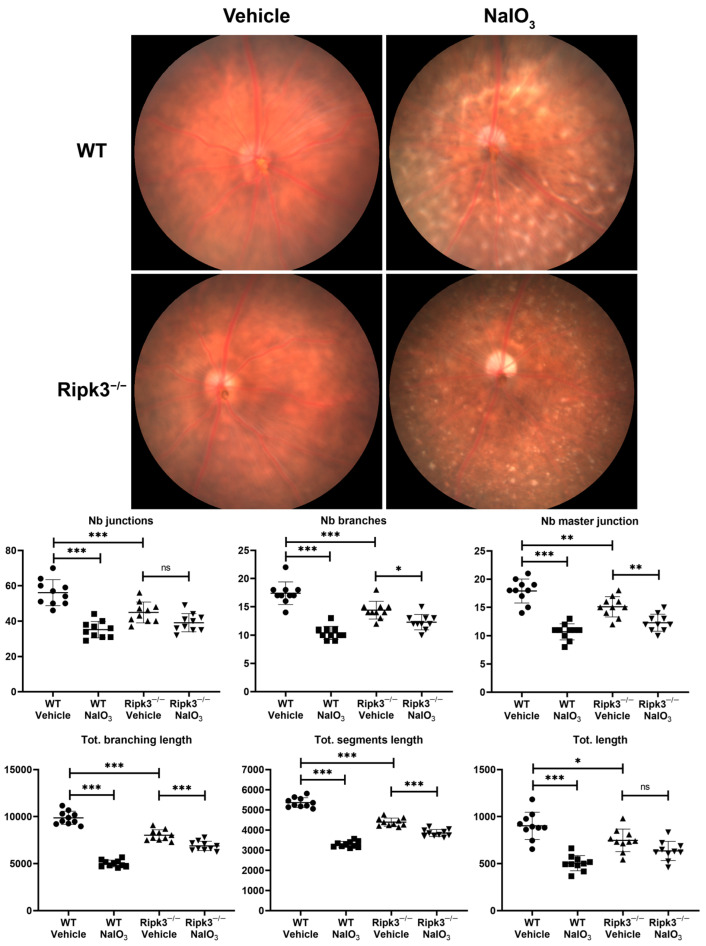
Decreased retinal microvascular branching in the absence of Ripk3. Fundus imaging (Micron III imaging system) was performed on WT and *Ripk3^−/−^* male and female mice (10 weeks) before and 1 week after receiving a single 50 mg/kg IP of NaIO_3_. Representative images are shown. To analyze retinal microvascular branching, the Canny Edge Detector and Angiogenesis Analyzer plug-in was used. The number (Nb) of junctions, branches, master junctions, and total (tot) branching length, segment length, and length are shown for WT and *Ripk3^−/−^* mice with a vehicle or NaIO_3_. (*** *p* < 0.001, ** *p* < 0.01, * *p* < 0.05, not significant (ns); *n* = 8.

To further examine the impact of Ripk3 deficiency on the retinal vasculature, we utilized non-invasive fundus imaging together with the Canny Edge Detector and Angiogenesis Analyzer plug-in. This allowed the analysis of branching characteristics of the retinal microvasculature for the number of junctions (multiple nodes together), branches (connected to vasculature by a single junction), and master junctions (junctions connecting at least two master segments) as well as the total branching length (a combination of all branch and segment lengths), the total segment length (length of extremities that have node on both ends) and total lengths (length of all segments) (Figure 2) [23,25]. Fundus images of retinas from *Ripk3*^−/−^ mice (10-week-old) demonstrated significantly decreased numbers of junctions, branches, master junctions, total length, total branching length, and total segment length (Figure 2), consistent with the reduced density of EC and PC.

Necroptosis plays a central role in RPE cell death [26], with a significant impact on outer retinal integrity and function and inner retinal and choroidal vasculature integrity as occurs during AMD pathogenesis. Since Ripk3 is involved in necroptosis, we next assessed the susceptibility of RPE loss with the NaIO_3_ treatment of *Ripk3*^−/−^ mice for retinal vasculature compared to their WT counterparts using fundus images. The mouse NaIO_3_ model is a well-established model for studying RPE cell loss and outer retinal degeneration, which occurs during the pathogenesis of dry AMD. WT mice treated with NaIO_3_ exhibited a dramatic decline in all retinal vascular parameters examined here, including junctions, branches, and master junction numbers, as well as total length, total branching length, and total segment length (Figure 2). In contrast, the treatment of *Ripk3*^−/−^ mice with NaIO_3_ tempered its impact with no significant additional changes in the total length and number of junctions. Although the number of branches, master junctions, total branching length, and total segment length decreased in NaIO_3_-treated *Ripk3*^−/−^ mice, the decrease was modest compared to that observed in WT mice. Thus, the increased Ripk3 level by NaIO_3_ or other stress stimuli could result in choroidal and retinal vascular degeneration and the pathogenesis of AMD.

**Figure 3:**
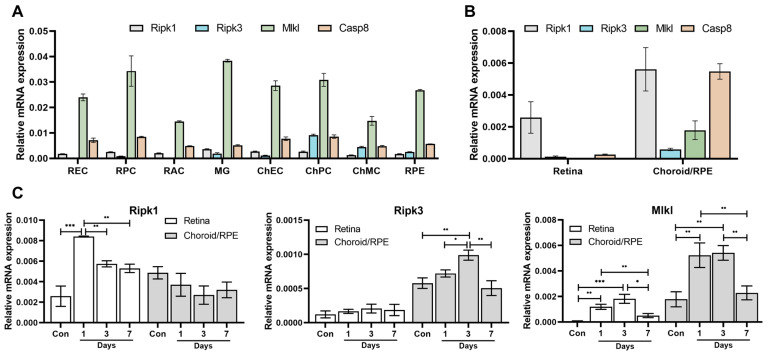
Ripk1, Ripk3, Mlkl, and Caspase 8 expression in retinal and RPE/choroid cells and tissues. (**A**) The expression of Ripk1, Ripk3, Mlkl, and Caspase 8 in various cell types, prepared from WT mouse retina and choroid in our laboratory, was determined by qPCR as described in the Materials and Methods. Please note the limited expression of Ripk3 in retinal EC (REC), RPC, and RAC. (**B**) The expression of Ripk1, Ripk3, Mlkl, and Caspase 8 in retina and choroid/RPE tissues prepared from 8-week-old WT mice. Please note that Ripk1 had a higher level of expression than Ripk3 or Mlkl. Please also note that choroid/RPE had significantly higher levels of Ripk1, Ripk3, Mlkl, and Casp8 than the retina. (**C**) The expression of Ripk1, Ripk3, and Mlkl in retina and choroid/RPE tissue prepared from WT mice treated with NaIO_3_. C57BL/6j male and female mice (8-week-old) received a single 50 mg/kg IP of NaIO_3_, and retina and choroid/RPE were harvested 1, 3, and 7 days later. The expressions of Ripk1, Ripk3, and Mlkl were then assessed by qPCR analysis as described in the Methods section. *** *p* < 0.001, ** *p* < 0.01, * *p* < 0.05; ANOVA, *n* = 8.

We next examined the expression of key players in necroptosis, namely Ripk1, Ripk3, Mlkl, and Caspase 8, in wild-type mouse retinal and choroidal cells, which we prepared in our laboratory. Figure 3A shows the expression of these genes in retinal EC (REC), pericytes (RPCs), astrocytes (RACs), and microglial cells (MGs), as well as choroidal EC (ChEC), pericytes (ChPCs), melanocytes ChMCs), and RPE cells. Ripk1 is more uniformly expressed in both retinal and choroidal cells. Ripk3 expression was very low in REC and RAC, consistent with previous reports [27], with ChPC expressing the highest levels. The expression of Mlkl was significantly higher than Ripk1 and Ripk3 in both retinal and choroidal cells, with the lowest expression in RAC and ChMC. Caspase 8 was also expressed more uniformly across the retinal and choroidal cells. Figure 3B shows the expression of these genes in whole retina tissue and the RPE/choroid complex prepared from WT mice. We observed the more predominant expression of these genes in the choroid compared with the retina. Thus, necroptosis may be more predominant in the choroid than the retina.

Given that NaIO_3_ could mediate the necroptosis of RPE cells and the degeneration of the outer retina, we next assessed its influence on Ripk1, Ripk3, and Mlkl expressions in the RPE/choroid and retina tissues following NaIO_3_ administration. Although the expression of necrosome components was more prominent in the choroid compared to the retina, as stated above, Ripk1 expression in the retina increased by 1 day following NaIO_3_ administration and then decreased (Figure 3C). There was no significant change in the expression of Ripk1 in the RPE/choroid following NaIO_3_ administration. We saw the opposite effect on Ripk3 expression, with no impact observed in the retina. However, NaIO_3_ administration resulted in a gradual significant increase in Ripk3 expression in the RPE/choroid by Day 3, which declined significantly by Day 7 to control levels. Mlkl expression in both the retina and RPE/choroid was affected following NaIO_3_ administration. Mlkl expression significantly increased on Days 1 and 3, then decreased to control levels on Day 7 following NaIO_3_ administration in both the retinal and RPE/choroid (Figure 3C). These results are consistent with the proposed role of NaIO_3_ in mediating RPE cell necroptosis and outer retinal degeneration.

### 3.3. Enhanced Retinal Neovascularization in Ripk3^−/−^ Mice During OIR

**Figure 4:**
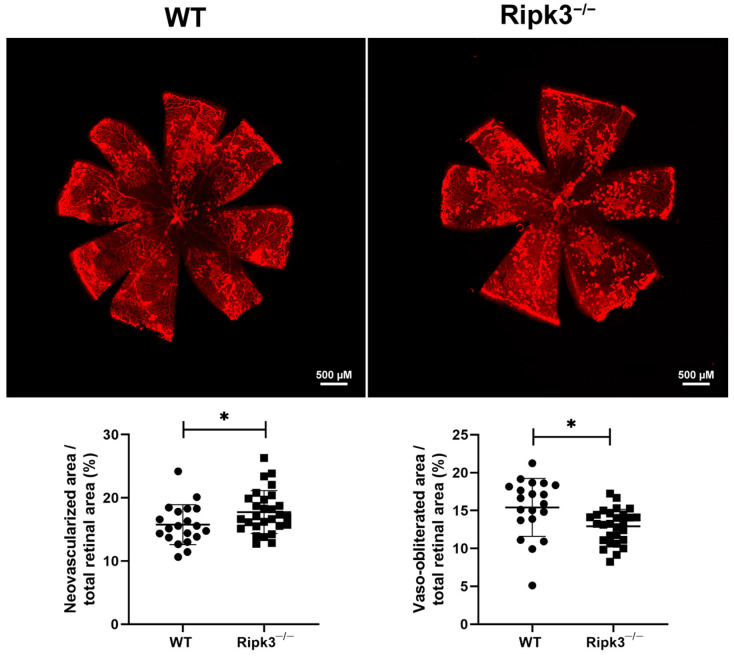
Hyperoxia-driven neovascularization is increased in *Ripk3*^−/−^ mice. Male and female mice were exposed to a cycle of hyperoxia and room air (OIR), as detailed in the Methods section. To visualize the vasculature, retinas in WT and *Ripk3*^−/−^ mice were wholemount stained with anti-collagen IV. At P17, the area of neovascularization and vaso-obliteration relative to the retina was quantified (*n* = 20, * *p* < 0.05). Please note increased neovascularization in *Ripk3*^−/−^ mice.

**Figure 5:**
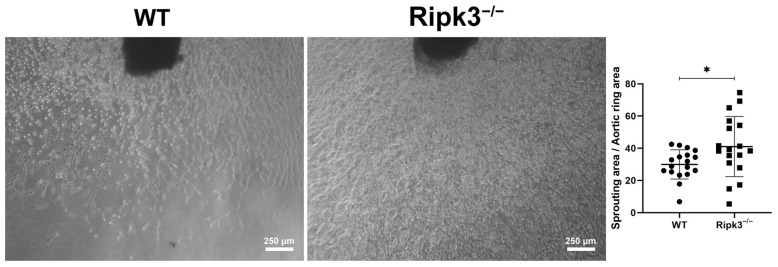
Increase thoracic aortic ring sprouting in the absence of Ripk3. Thoracic aortic rings, prepared from 4-week-old WT and *Ripk3*^−/−^ mice, were embedded in Matrigel, and 7 days later, photographed with the mean area of outgrowths quantified. (* *p* < 0.05; *n* = 18).

The developing retinal vasculature senses alterations in the level of oxygen and lays down the appropriate amount of vasculature to suit its needs. Thus, the developing retinal vasculature is vulnerable to changes in oxygen levels and is readily damaged with high oxygen exposure. During OIR, P7 mice are subjected to high oxygen (75%) for 5 days, followed by 5 days at room air (20% oxygen) [28]. Exposure to high oxygen mitigates the growth of additional blood vessels and promotes the loss of existing blood vessels. The retina becomes ischemic at room air, promoting aberrant neovascularization, which extends into the vitreous. Figure 4 demonstrates increased retinal neovascularization and a reduced area of vessel obliteration remaining at P17 in *Ripk3*^−/−^ mice compared to their WT counterparts. In addition, the utilization of an ex vivo aortic sprouting ring assay also demonstrated increased sprouting angiogenesis in the absence of Ripk3 (Figure 5). These data suggest a role for Ripk3 expression in modulating sprouting angiogenesis.

**Figure 6:**
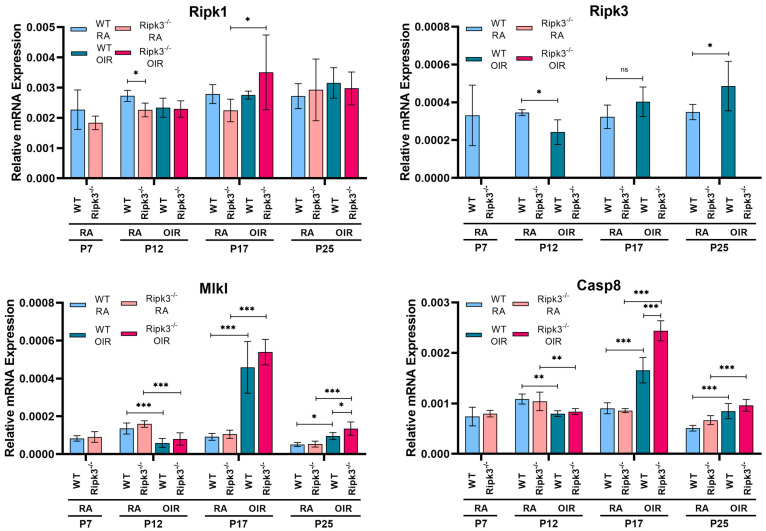
Expressions of Ripk1, Ripk3, Mlkl, and Casp8 during OIR. WT and *Ripk3*^−/−^ mice were subjected to OIR or maintained in room air, as detailed in the Methods section. RNA was prepared at the indicated time point, and expressions of Ripk1, Ripk3, Mlkl, and Casp 8 were determined by qPCR. (*** *p* < 0.001, ** *p* < 0.01, * *p* < 0.05; *n* = 6). ns = not significant.

We next asked whether exposure to hyperoxia followed by normoxia, which enhanced neovascularization, impacts the expression of necroptosis regulatory network genes, including Ripk1, Ripk3, Mlkl, and Caspase 8. Figure 6 shows the expression of these genes in retinas prepared from P7, P12, P17, and P25 WT and *Ripk3*^−/−^ mice reared in room air or subjected to OIR. Ripk1 was similarly expressed in retinas from P7 WT and *Ripk3*^−/−^ mice, and its expression was not impacted during OIR in the retinas of these mice. The Ripk3 expression level was 10-fold lower than Ripk1 in the retina. Its expression decreased with hyperoxia and, subsequently, at P25, increases in WT mice occurred during OIR. Mlkl expression was similar in WT and *Ripk3*^−/−^ mice, which did not change significantly up to P25 in room air-reared mice. However, its expression is similarly decreased with hyperoxia exposure (P12) and increased during ischemia-mediated neovascularization (P17), which reaches a normal level following the regression of newly formed vessels (P25). The Caspase 8 expression was similar in WT and *Ripk3*^−/−^ mice up to P25, and its expression was not affected by hyperoxia (P12). However, its expression was significantly increased during ischemia-mediated neovascularization in both WT and *Ripk3*^−/−^ mice (P17) but reached more normal levels following the regression of the newly formed vessels (P25).

### 3.4. Inflammation, CNV, and Fibrosis Are Enhanced in Ripk3^−/−^ Mice

**Figure 7:**
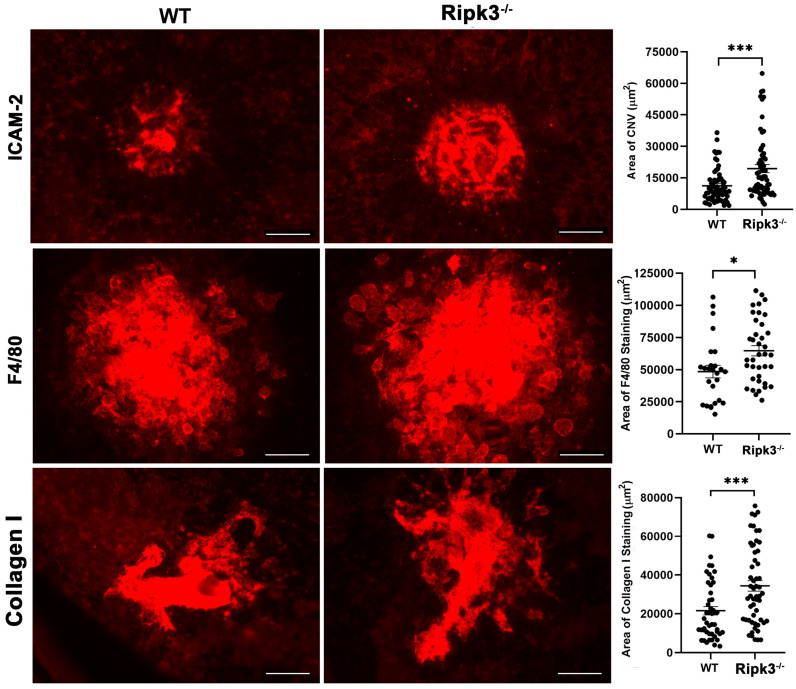
Increased ICAM-2, F4/80, and collagen I staining following laser photocoagulation in *Ripk3*^−/−^ mice. Male and female mice (3-month-old) underwent the laser photocoagulation-induced rupture of Bruch’s membrane. At the noted intervals following laser photocoagulation, the eyes were sectioned at the equator and the anterior half, with the vitreous and retina discarded. The remaining RPE/choroid complex was stained with anti-ICAM-2 (vascular marker at 2 weeks), anti-F4/80 (macrophage marker at 3 days), or anti-collagen I (fibrosis marker at 5 weeks). Images were taken from a Zeiss microscope (Zeiss, Chester, VA, USA) used in digital format. The total area (in µm^2^) was measured using ImageJ software (right panel). Each dot is one laser spot. Scale bar = 50 µm. All experiments were repeated with at least 8 mice. (* *p* < 0.05, *** *p* < 0.001; Student’s unpaired *t*-test (2-tailed).

**Figure 8:**
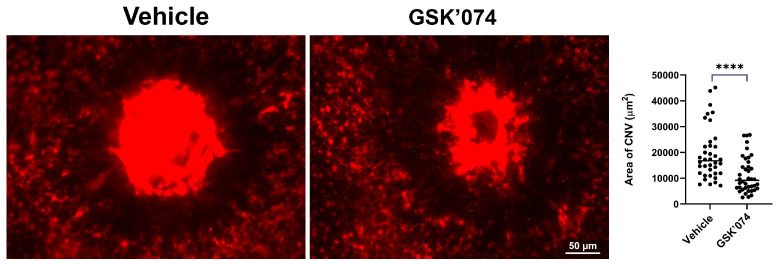
Inhibition of Ripk1 and Ripk3 activity mitigates choroidal neovascularization. Male and female mice (3-month-old) underwent the laser photocoagulation-induced rupture of Bruch’s membrane. The lasered mice were treated with a vehicle or our novel inhibitor of both Ripk1 and Ripk3 and compound GSK’074, as detailed in the Methods section. Areas of neovascularization were determined as detailed in the legend of Figure 7. Scale bar = 50 µm. All experiments were repeated with at least 8 mice. (**** *p* < 0.0001; Student’s unpaired *t*-test (2-tailed).

Choriocapillaris nourishes the outer retina, with defects in the circulation developing during nAMD pathogenesis. The role Ripk3 plays during pathological CNV is not completely delineated. Here, we determined whether Ripk3 expression influences macrophage infiltration, CNV, and fibrotic scar formation following the laser photocoagulation-induced rupture of Bruch’s membrane. *Rikp3*^−/−^ mice demonstrated increased CNV following laser photocoagulation compared to their WT counterparts (Figure 7). Next, we assessed whether increased CNV levels were attributable to enhanced macrophage recruitment. *Ripk3*^−/−^ choroid/RPE samples demonstrated increased F4/80 (a pan macrophage marker) immunostaining 3 days following laser photocoagulation (Figure 7). This correlated with increased fibrotic scar formation in the choroid/RPE, as shown by using anti-collagen I staining (Figure 7). We recently developed a novel inhibitor of Ripk1/3 activity [18]. We next asked whether Ripk1/3 activity is important during CNV by treating WT mice subjected to laser photocoagulation with the Ripk1/3 inhibitor, GSK’074. Figure 8 shows that the inhibition of Ripk1/3 activity by GSK’074 mitigated CNV. Thus, the expression and/or activity of Ripk3 may play an important role in the modulation of CNV and subretinal fibrosis.

## 4. Discussion

Necroptosis has similarities to both necrosis and apoptosis. Like necrosis, it requires energy with cell death, resulting in the loss of plasma membrane integrity and activation of the immune system, but it uses specific genes like apoptosis. While apoptosis plays a key role during organogenesis, as illustrated by devasting developmental defects when key apoptotic proteins are deleted [29,30,31], the role necroptosis plays is less understood. Here, we show that the global lack of Ripk3 expression impacted normal retinal vascularization prior to maturation, which persisted. We observed a similar initial rate of coverage from the expanding retinal superficial layer in P5 *Ripk3*^−/−^ mice compared to their WT counterparts. These results are in contrast with a study showing how mice lacking Ripk3 expression in EC exhibited decreased retinal vascular expansion at P5 but not at P4 or P6 [32]. Thus, the impact of Ripk3 expression on early postnatal retinal vascular expansion appears to be minimal. This notion is supported by the limited expression of Ripk3 in REC, RPC, and RAC, as well as whole retinas with and without NaIO_3_ treatment from wild-type mice, as demonstrated here.

The examination of retinal vasculature after the completion of primary vascular plexus in 3-week-old mice showed that the density of retinal vascular EC and PC is significantly decreased in the absence of Ripk3 despite the similar rate of early retinal vascular expansion. This could be attributed to the enhanced rate of apoptosis in the absence of Ripk3 during the early phase of vascular remodeling, which requires further verification. However, following retinal vasculature maturation in 6-week-old mice, retinal vascular EC and PC densities were similar in the presence or absence of Ripk3. Perhaps, given the decreased numbers of retinal vascular EC and PC in *Ripk3*^−/−^ mice, pruning was not warranted to maintain vascular homeostasis and oxygen needs. We previously noted a similar lack of pruning in other mice with low retinal vascular EC and PC densities at 3 weeks of age [21]. Thus, forgoing pruning may be a protective mechanism to maintain vascular homeostasis, preventing long-term ischemia and progressive tissue damage. However, fundus image analysis showed how significant abnormalities in various structural aspects of the retinal vascular network in *Ripk3*^−/−^ mice are present in older mice. In addition, *Ripk3*^−/−^ mice did not show additional retinal vascular abnormalities following exposure to NaIO_3_, as was noted in WT mice. This is consistent with the demonstrated role of Ripk3 in the necroptosis of RPE cells incubated with NaIO_3_ and the degeneration of the outer retina with a significant impact on retinal vasculature integrity in wild-type mice [26]. Thus, the Ripk3 expression, especially in EC, is essential for proper retinal vascularization [32], perhaps through the modulation of Caspase 8 expression and activity.

Necroptosis influences disease states through its ability to modulate cell death and inflammation. Two ocular models of neovascularization, the OIR (retinal) and the laser photocoagulation-induced rupture of Bruch’s membrane model (choroidal), were examined here. Retinal and choroidal neovascularization was enhanced in *Ripk3*^−/−^ mice. Following hyperoxia, retinal neovascularization is driven by the ischemic retina when mice are returned from hyperoxia to room air. Here, we observed a lack of Ripk3 expression-enhanced retinal neovascularization, perhaps as a result of increased Caspase 8 expression in response to ischemia. Our results contrast with those previously reported in mice where Ripk3 was deleted in microglia and subjected to OIR, showing reduced retinal neovascularization [27]. Unfortunately, the impact of Ripk3-targeted deletion in EC on retinal neovascularization during OIR was not previously assessed [32]. We also showed enhanced sprouting angiogenesis in aortas from *Ripk3*^−/−^ mice. In addition, our gene expression studies showed the low expression of necroptosis modulators in the retina compared to the choroid. Thus, necroptosis regulatory pathways may have variable roles in the retina compared with the choroid and perhaps other tissues.

Ripk1 and Ripk3 activities are noted as essential for CNV in wild-type mice in a Caspase activation-dependent manner, but the inhibition of necroptosis does not affect CNV in *Ripk3*^−/−^ mice [33]. Here, we observed increased neovascularization in the choroid of *Ripk3*^−/−^ mice following laser photocoagulation compared with wild-type mice. Increased CNV correlated with increased mononuclear phagocyte recruitment in *Ripk3*^−/−^ mice and subsequent fibrosis. Thus, in the absence of Ripk3, the modulation of inflammation and/or necrosis becomes dysregulated in the choroid, resulting in enhanced subretinal neovascularization and subsequent enhanced fibrosis. Our results are consistent with those demonstrated in Caspase 8-deficient mice, where the increased Ripk3 level results in reduced angiogenesis due to increased necroptosis, and the loss of macrophages results in reduced retinal neovascularization [34]. Thus, the expression of Caspase 8 is important in keeping Ripk3 availability in check, such that increased Ripk3 availability in the absence of Caspase 8 could mitigate neovascularization [34].

We found that Ripk1, Mlkl, and Caspase 8 expression in P7 *Ripk3*^−/−^ mice were like WT mice. Exposure to hyperoxia or ischemia during OIR did not affect Ripk1 expression in WT and *Ripk3*^−/−^ mice during OIR. In addition, the expression of Ripk3 in WT mice was not affected by hyperoxia or ischemia during OIR. In contrast, Mlkl, but not Caspase 8 expression, decreased with hyperoxia but increased with ischemia during OIR in both WT and *Ripk3*^−/−^ mice. Although Caspase 8 expression was not affected by hyperoxia, its expression was significantly increased, especially in *Ripk3*^−/−^ mice with ischemia and increased neovascularization during OIR. The increased retinal neovascularization noted in *Ripk3*^−/−^ is consistent with the results in EC-targeted Caspase 8-deficient mice showing reduced retinal neovascularization during OIR. Thus, increased Caspase 8 expression may be responsible for increased neovascularization. The increased levels of Ripk3 in the absence of Caspase 8 and the increased phosphorylation and activation of P38 MAP kinase result in aberrant VE-cadherin junctional localization and reduced neovascularization [34]. We did not observe significant differences in P38 levels in retinas prepared from WT and *Ripk3*^−/−^ mice (Supplementary Figure S1). Thus, Ripk3 expression and activity could similarly keep Caspase 8 expression and/or activity in check, which is a possibility that will benefit from further investigation.

Ripk3 knockdown in EC has been shown to be associated with decreased levels of myoferlin and enhanced turnover of growth factor receptors, including VEGFR2 [32]. This was concomitant with increased ERK activity and changes in EC characteristics toward an arterial (Dll4 and Notch4) and lymphatic (Sox18 and VEGFR3) identity [34]. The authors did not note these changes in early-developing retinal vasculature in vivo and proposed that these changes may occur in the more mature retinal vasculature of Ripk3-deficient EC. We examined the expression of MAP kinase ERKs, myoferlin, and arterial and lymphatic markers in retinas from WT and *Ripk3*^−/−^ mice. There were no significant changes noted in the expression of these markers in retinas from *Ripk3*^−/−^ compared to WT mice (Supplementary Figures S2–S4). The contrast between our findings in the retina compared to those reported in cultured EC could be attributed to the different sources of EC, human umbilical vein EC (HUVEC), and retinal vascular EC. However, the important role of Ripk3 and its interaction with the chaperon protein, such as myoferlin in EC, to protect the growth factor receptor from degeneration could also contribute to the delayed wound healing noted in *Ripk3*^−/−^ mice and the diminished response of *Ripk3*^−/−^ fibroblasts to TGFβ and PDGF [35].

**Figure 9:**
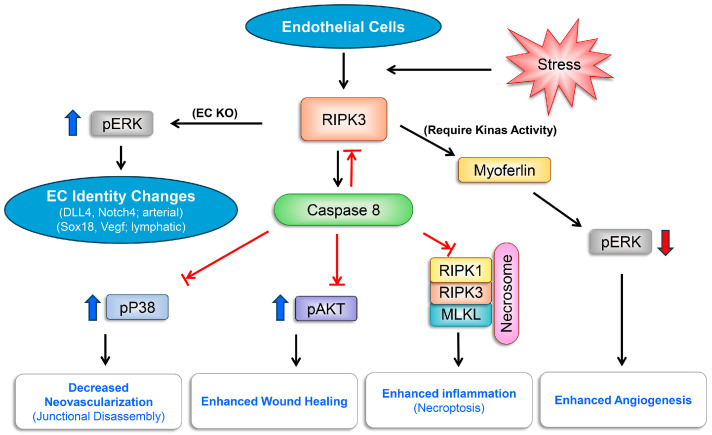
A summary of pathways engaged downstream of RIPK3 in vascular endothelial cells (ECs) following stress exposure and their impact on angioinflammatory activities and cell death-related processes.

The interrelationships among the pathways discussed are summarized in Figure 9. The higher expression of necroptosis regulatory protein in the choroid compared with the retina and their up-regulation with oxidative stress, such as treatment with NaIO_3_, as shown here, suggest their important role in the pathogenesis of AMD. Using a pharmacological inhibitor of Ripk1 and Ripk3 activity, we showed the significant attenuation of CNV in the mouse laser model. The cell-autonomous action and potential scaffold activities of these proteins in the modulation of necroptosis and/or apoptosis in the choroid and retina remain largely elusive. The further delineation of the role these proteins play in the modulation of choroidal function may aid in the development of new treatment strategies for both forms of AMD, including dry and wet forms, as well as other ocular diseases with vascular dysfunction.

The key players involved in the regulation of cell death pathways in response to various stress signals include RIPK1, RIPK3, MLKL, and Caspase 8. Interactions among these molecules, through highly regulated processes whose details remain largely elusive, impact apoptosis and necroptosis. Necroptosis is highly inflammatory and could be more prominent in the choroid. The activity and interactions of RIPK1, RIPK3, and Caspase 8 are important in the regulation of cell death, and angioinflammatory processes. The activity of RIPK3 is kept in check through interactions with Caspase 8, regulating cell death pathways, necroptosis and apoptosis. The global deletion of RIPK1 or Caspase 8 is embryonic and lethal, while *Ripk3*^−/−^ mice and dead kinase RIPK1 mice are viable. In addition, RIPK3 ablation restores all the defects in Caspase 8 deficiency. The targeted deletion of Caspase 8 in EC unleashes RIPK3 activity and increases P38 MAPK phosphorylation, driving junctional disassembly and the mitigation of retinal neovascularization [34]. Caspase 8’s interaction with RIPK3 influences necroptosis and inflammation [36]. In the necrosome, RIPK1 signals to RIPK3 to mediate necroptosis and enhance inflammation. However, RIPK3 signals to RIPK1 when the kinase activity of RIPK3 is inhibited to mediate apoptosis. The kinase activities of RIPK1 and RIPK3 are important in the modulation of angiogenesis. The inhibition of kinase activity of RIPK1 or RIPK3 mitigates choroidal neovascularization (CNV), but complete RIPK3 deficiency (*Ripk3*^−/−^) does not. Here, we showed enhanced retinal and choroidal neovascularization in *Ripk3*^−/−^ mice, as well as increased ex vivo aortic sprouting. The studies with EC targeted the deletion of RIPK3 and showed abnormal retinal vascularization at p5 but not P4 or P6 [32]. We did not detect significant differences in the spreading of developing retinal vasculature in P5 retinas from *Ripk3*^−/−^ mice. Unfortunately, retinal or choroidal neovascularization was not assessed in the EC-targeted Ripk3-deficient mice. The vascular changes noted in these mice were attributed to the decreased level of myoferlin, a chaperone protein whose interaction with RIPK3 protects growth factor receptor degradation, including VEGFR2, and may increase ERK activity, altering EC identity and abnormal retinal vascular sprouting and branching morphogenesis. *Ripk3*^−/−^ mice also exhibited delayed wound healing [35]. This was attributed to decreased levels of TGFβ and PDGF receptor expressions and the failure of *Ripk3*^−/−^ fibroblasts to migrate in response to TGFβ or PDGF, as well as changes in angioinflammatory activities. Increased RIPK3 expression in fibroblasts results in increased AKT activity and enhanced wound healing. Thus, the interaction of RIPK3 with a chaperon protein could regulate growth factor receptor degradation in fibroblast and proper wound healing. We observed a significant decrease in the density of retinal ECs and pericytes in 3-week-old *Ripk3*^−/−^ mice that matched that of mature vasculature in 6-week-old wild-type mice, suggesting abnormal pruning and remodeling in the mature retinal vasculature of *Ripk3*^−/−^ mice, which was further confirmed from the assessment of fundus images. However, the examination of retinal tissues at different postnatal days and during OIR did not show any differences in the levels of myoferlin and ERK, P38, and AKT activation, as well as the expression of EC markers (Arterial vs. lymphatic) in *Ripk3*^−/−^ mice. These differences between *Ripk3*^−/−^ and EC-targeted RIPK3-deficient mice could be attributed to the complete absence of RIPK3 rather than just EC. However, the EC expression of RIPK3 appears to have a significant impact on the properties of EC, including proliferation, migration, and sprouting, perhaps through interactions with Caspase 8. The targeting of RIPK3 in microglia also mitigates retinal neovascularization due to reduced levels of FGF2 [27]. Many of the mechanistic studies have been performed in HUVEC, which are very different ECs from retinal and choroidal ECs examined here and need further verification. In the future, the investigation of retinal and choroidal tissue single-cell RNA sequencing should provide further clarification of the expression of RIPK3 in different cell types and their coordinated responses to stress challenges impacting angioinflammatory processes in a tissue-specific manner. However, the preferential expression of the regulatory proteins discussed here in the choroid suggests an important role for these pathways in the modulation of inflammatory processes in the choroid.

## 5. Conclusions

The studies presented here support the important role of Ripk3 during vascular development and neovascularization. We showed that the initial rate of spreading of the retinal vasculature is not significantly impacted by Ripk3 deficiency at P5. However, the densities of retinal vascular ECs and PCs were significantly lower in the retinas of 3-week-old *Ripk3^−/−^* mice following the formation of the primary retinal vascular plexus. The densities of vascular cells were not significantly different in *Ripk3^−/−^* mice compared to wild-type mice at 6 weeks of age, suggesting the mitigation of pruning and remodeling, which normally occurs in wild-type mice. We propose that this is due to the formation of an insufficient primary vascular plexus in *Ripk3^−/−^* mice, which is inherently spared from further pruning and remodeling. The changes in vascular densities are consistent with noted structural and branching defects in mature *Ripk3^−/−^* mice in fundus images. Similar vascular abnormalities were suggested in EC-targeted Ripk3-deficient mice based on in vitro knockdown studies in HUVEC, linking potential Ripk3 interactions with myoferlin, a chaperon protein, and the protection of growth factor receptors from degradation, such as that in the absence of the Ripk3-enhanced degradation of growth factor receptor(s), was noted. These changes may contribute to insufficient angiogenesis along with the regulation of ERK activity and changes in EC identity. We did not note any changes in the levels of myoferlin, P38, ERK, and EC identity gene expression in retinas from *Ripk3^−/−^* mice compared to wild-type mice.

Although retinal and choroidal neovascularization was not assessed in EC-targeted Ripk3-deficient mice, we found that *Ripk3^−/−^* showed enhanced retinal and choroidal neovascularization. We also noted enhanced sprouting in aortic tissues prepared from *Ripk3^−/−^* mice. Our gene expression studies of retinas prepared from mice reared at room temperature compared to those from mice subjected to OIR showed a significant up-regulation of Caspase 8 expression in *Ripk3^−/−^* mice. Although Caspase 8 is well recognized as a key regulator of Ripk3 activity, the importance of Ripk3 expression on Caspase 8 activity is limited. Thus, the global lack of Ripk3 may result in increased Caspase 8 activity and the increased ischemia-mediated neovascularization noted here. This is consistent with reduced ischemia-mediated neovascularization in EC-targeted Caspase 8-deficient mice. Thus, the further investigation of Ripk3’s impact on Caspase 8 activity, not only in vascular ECs but also PCs, will help to better delineate the role of Ripk3 in the modulation of cell death pathways and its impact on developmental and pathological angiogenesis.

## Figures and Tables

**Table 1:** Retinal vascular cell numbers.

Cell Types	Age	WT	*Ripk3* ^−/−^
Pericytes (PCs)	P21	43.53 ± 1.32	26 ± 0.75 ****
Endothelial Cell (EC)	P21	136.2 ± 3.67	124.3 ± 1.46 **
Pericytes (PCs)	P42	27.98 ± 0.78	28.85 ± 0.73
Endothelial Cell (EC)	P42	121.9 ± 1.71	124.4 ± 1.68

Number of cells per high-power field at (×400). The p-values were calculated by comparing samples from WT to *Ripk3*^−/−^ mice at the postnatal ages (days) noted as P21 and P42: **** *p* < 0.0001; ** *p* < 0.01.

## Data Availability

All the data are included in the manuscript and supplementary file.

## Supplementary Materials

The following supporting information can be downloaded at https://www.mdpi.com/article/10.3390/cells13242109/s1. Figure S1: Myoferlin expression; Figure S2: MAPK/ERK expressions; Figure S3: P38 MAPK expression; Figure S4: Arterial and lymphatic markers expression; Table S1: Detailed statistical analysis of the data.
