# Supplementary material for: Receptor-Interacting Protein Kinase-3 Expression Impacts Ocular Vascular Development and Pathological Neovascularization
Source: Cells. 2024 Dec 20;13(24):2109. doi: 10.3390/cells13242109 (PMC11726843; doi:10.3390/cells13242109)
Supplement: Supplementary file 1 [file cells-13-02109-s001.zip › Supplementary file_1018.24.pdf]

Supplementary Materials and Methods

Western Blot Analysis

Retinas were harvested from WT or *Ripk3*$^{-/-}$ male and female mice at 3 weeks of age or postnatal day 10, and immediately snap-frozen at −80°C. Two retinas from each mouse were homogenized in 200 mL of lysis buffer (142.5 mM KCl, 5 mM $MgCl_2$, 10 mM HEPES, 1% NP-40, 1 mM Na_3VO_4, 1 mM NaF), supplemented with complete protease inhibitor cocktail (Roche Applied Sciences, Mannheim, Germany; 11836153001). The homogenates were then subjected to brief sonication and centrifugation at 15,000 ×g for 15 min. Protein concentrations of the lysates were quantified using the BCA protein assay (ThermoFisher Scientific, Carlsbad, CA, USA; 23227). Lysates were adjusted for protein content and mixed with the appropriate volume of 6× SDS loading buffer. Equal amounts of protein (40 μg per sample) were separated by SDS-PAGE using 4–20% Tris-glycine gels (ThermoFisher Scientific; XP04200BOX) and transferred onto a nitrocellulose membrane. The membrane was blocked with 5% skim milk in TBS (Tris-buffered saline; 20 mM Tris-HCl, 150 mM NaCl, pH 7.6) containing 0.05% Tween 20, followed by incubation with the appropriate primary antibodies, including anti-Myoferlin (PA590458) and anti-β-actin (MA515739) (ThermoFisher Scientific), as well as anti-phospho-ERK (9106S), anti-ERK (9102S), anti-phospho-P38 (9211S), and anti-P38 (9212S) all from Cell Signaling (Danvers, MA, USA). Subsequent incubation with appropriate HRP-conjugated secondary antibodies (JacksonImmuno, Hershey, PA, USA) was performed, and the protein bands were visualized using the ECL kit (Sigma, St. Louis, USA; Cytiva RPN2209,). The band intensities were quantified using ImageJ software (NIH).

RNA Extraction and Real-Time Quantitative PCR (qPCR)

Total RNA from mouse retinas was isolated using Trizol reagent (ThermoFisher Scientific; 15596018;) and RNeasy Mini Kit (Qiagen, Maryland, CA USA; 74104) was used for purification. Total RNA (1 μg) was used to synthesize cDNA with RNA to cDNA EcoDry Pre-mix (Double Primed) (Takara Bio USA, Inc., San Francisco, CA, USA; 639549;). The cDNA samples were diluted 1:10 and used as the templates for qPCR analysis performed in QuantStudio 3 Real-Time PCR Systems (ThermoFisher Scientific) with TB-Green Advantage qPCR Premix (Takara Bio; 639676). The mouse primers used in this study are as follows: Dll4 forward: 5′- aagaatagcggcagtggtcg′-3′, Dll4 reverse: 5′-gatgagagagtttcctggcg-3′, Notch4 forward: 5′-ggagtgtctctttgatggctacg-3′, Notch4 reverse: 5′-gccacattcagcgttattgc-3′, Sox18 forward: 5′-gcact ggccaaactaggtc-3′, Sox18 reverse: 5′-tgtaatagaccgcgctgcta-3′, Flt4/Vegfr3 forward: 5′-ccatctc aacgtggtcaacc-3′, Flt4/Vegfr3 reverse: 5′-aagttggagaggttgccgta-3′.

Supplementary Figures

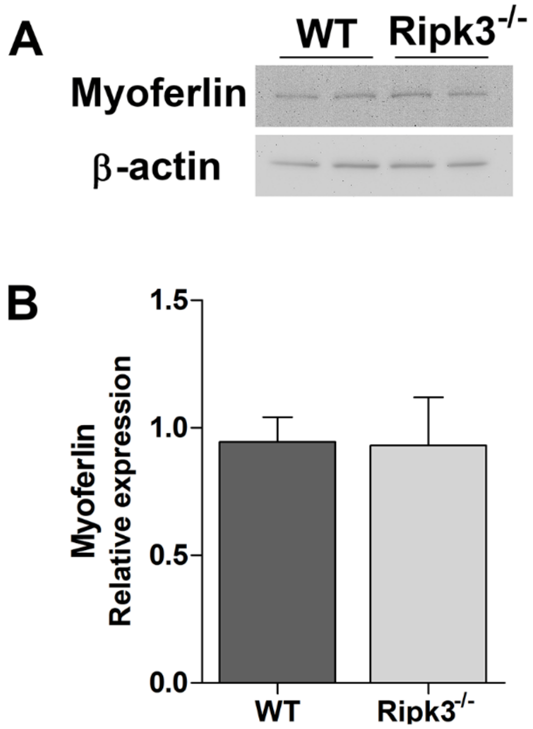

Figure S1. Western blot analysis of retinal lysates prepared from WT and *Ripk3*⁻/⁻ mice at 3 weeks of age (n = 2 mice for each genotype). (A) The levels of Myoferlin and β-actin were determined by Western analysis as detailed in Supplementary Methods. (B**)** Relative protein levels were quantified by measuring band intensities compared with β-actin.

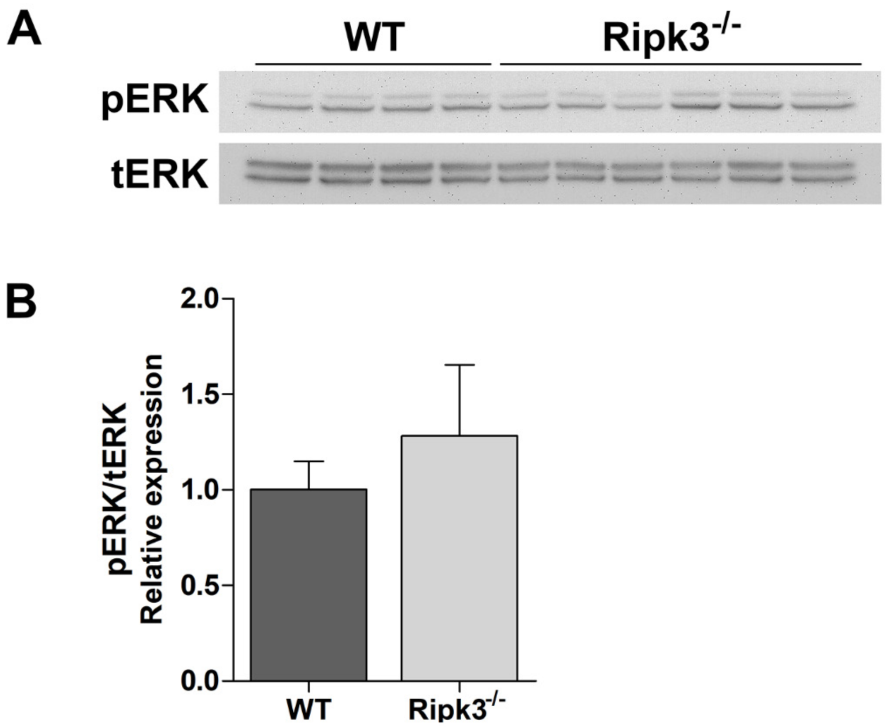

Figure S2. Western blot analysis was used to assess the active and total levels of ERK in retinal lysates prepared from WT and *Ripk3*$^{-/-}$ mice at postnatal day 10 (n = 4 for WT and n = 6 for *Ripk3* $^{-/-}$). (A) The levels of phosphorylated ERK and total ERK were determined as detailed in the supplementary Methods. (B) The relative level of activated ERK (phosphorylated) was assessed by quantifying band intensities relative to total level of protein.

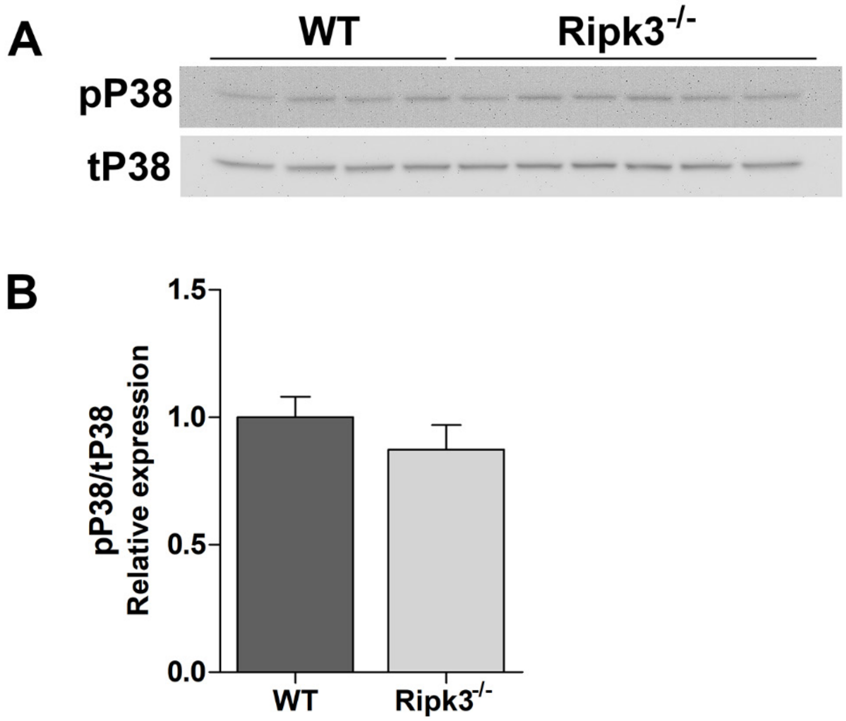

Figure S3. Western blot analysis was used to assess the active and total levels of P38 in retinal lysates prepared from WT and *Ripk3*$^{-/-}$ mice at postnatal day 10 (n = 4 for WT and n = 6 for *Ripk3* $^{-/-}$). (A) The levels of phosphorylated P38 and total P38 were visualized as detailed in the supplementary Method. (B) The relative level of activated (phosphorylated) P38 was assessed by quantifying band intensities compared with total P38.

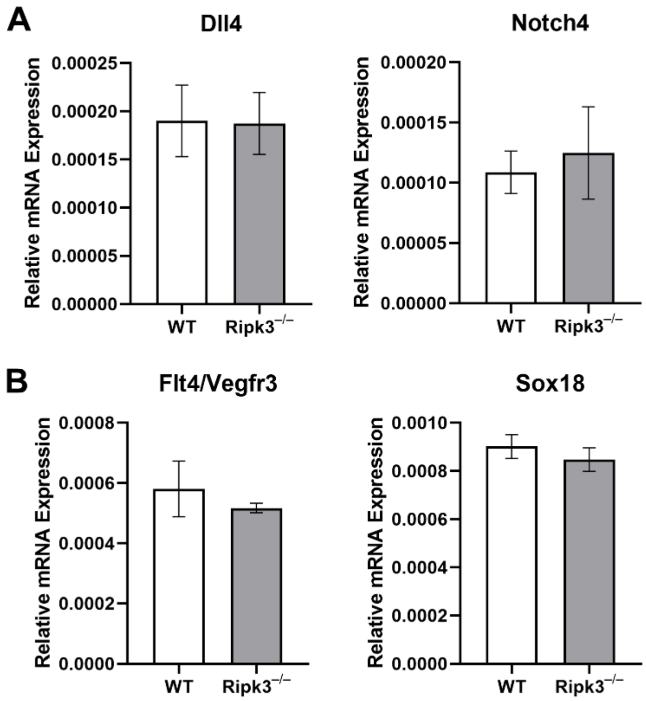

Figure S4. Dll4, Notch4, Flt4/Vegfr3, and Sox18 expression in P25 retinas. (A) Expression levels of arterial markers (Dll4 and Notch4) in WT and *Ripk3*$^{-/-}$ mouse retinas were determined by qPCR analysis (n = 3). (B) Expression levels of lymphatic markers (Flt4/Vegfr3 and Sox18) in WT and *Ripk*3$^{-/-}$ mouse retinas at P25 were assessed by qPCR analysis (n = 3).
