# Supplementary material for: Receptor-Interacting Protein Kinase-3 Expression Impacts Ocular Vascular Development and Pathological Neovascularization
Source: Cells. 2024 Dec 20;13(24):2109. doi: 10.3390/cells13242109 (PMC11726843; doi:10.3390/cells13242109)
Supplement: Supplementary file 1 [file cells-13-02109-s001.zip › Table S1_Ripk3 statistics summary.pdf]

Figure 1. Ripk3$^{-/-}$ mice demonstrate similar rate of expansion in the superficial layer.

Parametric and unpaired t-test was used

1. Vessel distance at P5

	WT	Ripk3$^{-/-}$
Mean	60.91	60.25
Standard deviation	5.021	5.010
P-value		0.6639

2. Tip cell counts

	WT	Ripk3$^{-/-}$
Mean	24.21	24.57
Standard deviation	3.623	3.084
P-value		0.7016

Table 1. Retinal Vascular Cell Numbers

Parametric and unpaired t-test was used

1. P21 Pericyte numbers

	WT	$Ripk3^{-/-}$
Mean	43.53	26
Standard deviation	1.32	0.75
P-value		<0.0001

2. P21 Endothelial cell numbers

	WT	$Ripk3^{-/-}$
Mean	136.2	124.3
Standard deviation	3.67	1.46
P-value		<0.001

3. P42 Pericyte numbers

	WT	$Ripk3^{-/-}$
Mean	27.98	28.85
Standard deviation	0.78	0.73
P-value		0.415

4. P42 Endothelial cell numbers

	WT	$Ripk3^{-/-}$
Mean	121.9	124.4
Standard deviation	1.71	1.68
P-value		0.305

Figure 2. Decreased retinal microvascular branching in the absence of Ripk3

One-way ANOVA with Tukey's multiple comparisons test was used

1. Number of Junctions

	WT Vehicle	WT $NaIO_3$	Ripk3$^{-/-}$ Vehicle	Ripk3$^{-/-}$ $NaIO_3$
Mean	56.1	35.2	44.9	39.1
Standard deviation	7.355	4.686	5.82	5.087

Tukey's multiple comparisons test	P-value
WT Vehicle vs. WT $NaIO_3$	<0.0001
WT Vehicle vs. Ripk3 Vehicle	0.0007
WT Vehicle vs. Ripk3 $NaIO_3$	<0.0001
WT $NaIO_3$ vs. Ripk3 Vehicle	0.0036
WT $NaIO_3$ vs. Ripk3 $NaIO_3$	0.45
Ripk3 Vehicle vs. Ripk3 $NaIO_3$	0.1356

2. Number of branches

	WT Vehicle	WT $NaIO_3$	Ripk3$^{-/-}$ Vehicle	Ripk3$^{-/-}$ $NaIO_3$
Mean	17.4	10.4	14.4	12.3
Standard deviation	2.011	1.174	1.578	1.337

Tukey's multiple comparisons test	P-value
WT Vehicle vs. WT $NaIO_3$	<0.0001
WT Vehicle vs. Ripk3 Vehicle	0.0007
WT Vehicle vs. Ripk3 $NaIO_3$	<0.0001
WT $NaIO_3$ vs. Ripk3 Vehicle	<0.0001
WT $NaIO_3$ vs. Ripk3 $NaIO_3$	0.0461
Ripk3 Vehicle vs. Ripk3 $NaIO_3$	0.0232

3. Number of master junctions

	WT Vehicle	WT $NaIO_3$	Ripk3$^{-/-}$ Vehicle	Ripk3$^{-/-}$ $NaIO_3$
Mean	17.9	10.7	15.1	12.3
Standard deviation	2.132	1.418	1.792	1.494

Tukey's multiple comparisons test	P-value

WT Vehicle vs. WT $NaIO_3$	<0.0001
WT Vehicle vs. Ripk3 Vehicle	0.0048
WT Vehicle vs. Ripk3 $NaIO_3$	<0.0001
WT $NaIO_3$ vs. Ripk3 Vehicle	0.0012
WT $NaIO_3$ vs. Ripk3 $NaIO_3$	0.5242
Ripk3 Vehicle vs. Ripk3 $NaIO_3$	0.0443

4. Total branching length

	WT Vehicle	WT $NaIO_3$	$Ripk3^{-/-}$ Vehicle	$Ripk3^{-/-}$ $NaIO_3$
Mean	9865	5014	8025	6885
Standard deviation	711.1	361.3	578.4	496.3

Tukey's multiple comparisons test	P-value
WT Vehicle vs. WT $NaIO_3$	<0.0001
WT Vehicle vs. Ripk3 Vehicle	<0.0001
WT Vehicle vs. Ripk3 $NaIO_3$	<0.0001
WT $NaIO_3$ vs. Ripk3 Vehicle	<0.0001
WT $NaIO_3$ vs. Ripk3 $NaIO_3$	<0.0001
Ripk3 Vehicle vs. Ripk3 $NaIO_3$	0.0003

5. Total segments length

	WT Vehicle	WT $NaIO_3$	$Ripk3^{-/-}$ Vehicle	$Ripk3^{-/-}$ $NaIO_3$
Mean	5363	3290	4392	3853
Standard deviation	244.4	149.9	200.2	175.7

Tukey's multiple comparisons test	P-value
WT Vehicle vs. WT $NaIO_3$	<0.0001
WT Vehicle vs. Ripk3 Vehicle	<0.0001
WT Vehicle vs. Ripk3 $NaIO_3$	<0.0001
WT $NaIO_3$ vs. Ripk3 Vehicle	<0.0001
WT $NaIO_3$ vs. Ripk3 $NaIO_3$	<0.0001
Ripk3 Vehicle vs. Ripk3 $NaIO_3$	<0.0001

6. Total length

	WT Vehicle	WT $NaIO_3$	$Ripk3^{-/-}$ Vehicle	$Ripk3^{-/-}$ $NaIO_3$

Mean	902.1	505	747.5	635.1
Standard deviation	144.6	81	119.8	102

Tukey's multiple comparisons test	P-value
WT Vehicle vs. WT $NaIO_3$	<0.0001
WT Vehicle vs. Ripk3 Vehicle	0.0226
WT Vehicle vs. Ripk3 $NaIO_3$	<0.0001
WT $NaIO_3$ vs. Ripk3 Vehicle	0.0002
WT $NaIO_3$ vs. Ripk3 $NaIO_3$	0.0696
Ripk3 Vehicle vs. Ripk3 $NaIO_3$	0.1428

Figure 3. Figure 3: Ripk1, Ripk3, Mlkl, and caspase 8 expression in retinal and RPE/choroid cells 294 and tissues.

One-way ANOVA with Tukey’s multiple comparisons test was used

1. qPCR for Ripk1

a. Ripk1 (Retina)

	Retina Control	Retina Day 1	Retina Day 3	Retina Day 7
Mean	2.58E-03	8.41E-03	5.74E-03	5.30E-03
Standard deviation	9.86E-04	3.54E-05	3.03E-04	4.05E-04

Tukey's multiple comparisons test	P-value
Control vs. Day 1	<0.0001
Control vs. Day 3	0.0014
Control vs. Day 7	0.0034
Day 1 vs. Day 3	0.0071
Day 1 vs. Day 7	0.003
Day 3 vs. Day 7	0.8007

b. Ripk1 (RPE/Choroid)

	RPE/Cho Control	RPE/Cho Day 1	RPE/Cho Day 3	RPE/Cho Day 7
Mean	4.87E-03	3.70E-03	2.69E-03	3.20E-03
Standard deviation	6.01E-04	1.12E-03	8.92E-04	7.60E-04

Tukey's multiple comparisons test	P-value
Control vs. Day 1	0.5207
Control vs. Day 3	0.1164
Control vs. Day 7	0.2589
Day 1 vs. Day 3	0.5485
Day 1 vs. Day 7	0.8997
Day 3 vs. Day 7	0.8963

2. qPCR for Ripk3

a. Ripk3 (Retina)

	Retina Control	Retina Day 1	Retina Day 3	Retina Day 7

Mean	1.22E-04	1.65E-04	2.07E-04	1.85E-04
Standard deviation	5.13E-05	3.06E-05	6.48E-05	8.27E-05

Tukey's multiple comparisons test	P-value
Control vs. Day 1	0.8636
Control vs. Day 3	0.4806
Control vs. Day 7	0.684
Day 1 vs. Day 3	0.8429
Day 1 vs. Day 7	0.977
Day 3 vs. Day 7	0.9727

b. Ripk3 (RPE/Choroid)

	RPE/Cho Control	RPE/Cho Day 1	RPE/Cho Day 3	RPE/Cho Day 7
Standard deviation	7.64E-05	5.26E-05	7.35E-05	1.08E-04

Tukey's multiple comparisons test	P-value
Control vs. Day 1	0.1975
Control vs. Day 3	0.0041
Control vs. Day 7	0.7291
Day 1 vs. Day 3	0.0314
Day 1 vs. Day 7	0.0749
Day 3 vs. Day 7	0.0028

3. qPCR for Mlkl

a. Mlkl (Retina)

	Retina Control	Retina Day 1	Retina Day 3	Retina Day 7
Mean	7.92E-05	1.19E-03	1.82E-03	5.06E-04
Standard deviation	1.34E-05	2.00E-04	3.52E-04	1.65E-04

Tukey's multiple comparisons test	P-value
Control vs. Day 1	0.0052
Control vs. Day 3	0.0004
Control vs. Day 7	0.2737
Day 1 vs. Day 3	0.0518
Day 1 vs. Day 7	0.0344
Day 3 vs. Day 7	0.001

b. Mlkl (RPE/Choroid)

	RPE/Cho Control	RPE/Cho Day 1	RPE/Cho Day 3	RPE/Cho Day 7
Mean	1.78E-03	5.23E-03	5.42E-03	2.28E-03
Standard deviation	5.88E-04	9.62E-04	5.73E-04	5.44E-04

Tukey's multiple comparisons test	P-value
Control vs. Day 1	0.0052
Control vs. Day 3	0.0004
Control vs. Day 7	0.2737
Day 1 vs. Day 3	0.0518
Day 1 vs. Day 7	0.0344
Day 3 vs. Day 7	0.001

Figure 4. Hyperoxia-driven neovascularization is increased in Ripk3$^{-/-}$ mice

Parametric and unpaired t-test was used

1. Neovascularization

	WT	Ripk3$^{-/-}$
Mean	15.76	17.75
Standard deviation	3.166	3.382
P-value		0.0432

2. Vaso-obliteration

	WT	Ripk3$^{-/-}$
Mean	15.42	12.92
Standard deviation	3.824	2.243
P-value		0.0142

Figure 5. Increase thoracic aortic ring sprouting in absence of Ripk3

Parametric and unpaired t-test was used

	WT	$Ripk3^{-/-}$
Mean	29.98	41.05
Standard deviation	9.053	18.69
P-value		0.0328

Figure 6. Expression of Ripk1, Ripk3, Mlkl, and Casp8 during OIR.

1. Ripk1 qPCR

For P7 samples, parametric and unpaired t-test was used

For P12, P17, and P25 samples, One-way ANOVA with Tukey's multiple comparisons test was used

	P7				P12			
	RA		OIR		RA		OIR	
	WT	Ripk3	WT	Ripk3	WT	Ripk3	WT	Ripk3
Mean	0.002272	0.001835	-	-	0.002728	0.002263	0.002338	0.002294
Stdev	0.000652	0.000224	-	-	0.000183	0.000227	0.000314	0.000270

	P17				P25			
	RA		OIR		RA		OIR	
	WT	Ripk3	WT	Ripk3	WT	Ripk3	WT	Ripk3
Mean	0.002786	0.002246	0.002754	0.003506	0.002718	0.002926	0.003157	0.002975
Stdev	0.000311	0.000373	0.000132	0.001237	0.000413	0.001022	0.000504	0.000544

Statistical analysis for P7 samples

t-test	WT	Ripk3$^{-/-}$
P-value		0.1704

Statistical analysis for P12 samples

Tukey's multiple comparisons test	P-value
WT RA vs. Ripk3$^{-/-}$ RA	0.0225
WT RA vs. WT OIR	0.0655
WT RA vs. Ripk3$^{-/-}$ OIR	0.0355
Ripk3$^{-/-}$ RA vs. WT OIR	0.9552
Ripk3$^{-/-}$ RA vs. Ripk3$^{-/-}$ OIR	0.9964
WT OIR vs. Ripk3$^{-/-}$ OIR	0.9905

Statistical analysis for P17 samples

Tukey's multiple comparisons test	P-value
WT RA vs. Ripk3$^{-/-}$ RA	0.5141
WT RA vs. WT OIR	0.9998
WT RA vs. Ripk3$^{-/-}$ OIR	0.2722
Ripk3$^{-/-}$ RA vs. WT OIR	0.562

Ripk3$^{-/-}$ RA vs. Ripk3$^{-/-}$ OIR	0.0185
WT OIR vs. Ripk3$^{-/-}$ OIR	0.2399

Statistical analysis for P25 samples

Tukey's multiple comparisons test	P-value
WT RA vs. Ripk3$^{-/-}$ RA	0.9475
WT RA vs. WT OIR	0.6678
WT RA vs. Ripk3$^{-/-}$ OIR	0.9072
Ripk3$^{-/-}$ RA vs. WT OIR	0.9305
Ripk3$^{-/-}$ RA vs. Ripk3$^{-/-}$ OIR	0.9992
WT OIR vs. Ripk3$^{-/-}$ OIR	0.964

2. Ripk3 qPCR

Parametric and unpaired t-test was used

	P7				P12			
	RA		OIR		RA		OIR	
	WT	Ripk3	WT	Ripk3	WT	Ripk3	WT	Ripk3
Mean	0.000331	2.26E-07	-	-	0.000345	0	0.000242	3.93E-07
Stdev	0.000161	4.49E-07	-	-	0.000015	0	0.000065	6.09E-07

	P17				P25			
	RA		OIR		RA		OIR	
	WT	Ripk3	WT	Ripk3	WT	Ripk3	WT	Ripk3
Mean	0.000323	2.84E-07	0.000403	1.49E-06	0.000348	4.15E-08	0.000486	0
Stdev	0.000062	5.71E-07	0.000078	3.66E-06	0.000040	1.02E-07	0.000131	0

t-test	P-value
P12 WT RA vs. WT OIR	0.0106
P17 WT RA vs. WT OIR	0.0921
P25 WT RA vs. WT OIR	0.0498

3. Mlkl qPCR

For P7 samples, parametric and unpaired t-test was used

For P12, P17, and P25 samples, One-way ANOVA with Tukey’s multiple comparisons test was used

	P7				P12			
	RA		OIR		RA		OIR	
	WT	Ripk3	WT	Ripk3	WT	Ripk3	WT	Ripk3
Mean	0.0000832	0.0000911	-	-	0.0001354	0.0001593	0.0000593	0.0000804
Stdev	0.0000144	0.0000279	-	-	0.0000291	0.0000172	0.0000239	0.0000331

	P17				P25			
	RA		OIR		RA		OIR	
	WT	Ripk3	WT	Ripk3	WT	Ripk3	WT	Ripk3
Mean	0.0000918	0.0001053	0.0004588	0.0005397	0.0000510	0.0000534	0.0000955	0.0001351
Stdev	0.0000178	0.0000216	0.0001359	0.0000668	0.0000110	0.0000157	0.0000186	0.0000348

Statistical analysis for P7 samples

t-test	WT	Ripk3$^{-/-}$
P-value		0.5579

Statistical analysis for P12 samples

Tukey's multiple comparisons test	P-value
WT RA vs. Ripk3$^{-/-}$ RA	0.4289
WT RA vs. WT OIR	0.0004
WT RA vs. Ripk3$^{-/-}$ OIR	0.0086
Ripk3$^{-/-}$ RA vs. WT OIR	<0.0001
Ripk3$^{-/-}$ RA vs. Ripk3$^{-/-}$ OIR	0.0003
WT OIR vs. Ripk3$^{-/-}$ OIR	0.5283

Statistical analysis for P17 samples

Tukey's multiple comparisons test	P-value
WT RA vs. Ripk3$^{-/-}$ RA	0.9899
WT RA vs. WT OIR	<0.0001
WT RA vs. Ripk3$^{-/-}$ OIR	<0.0001
Ripk3$^{-/-}$ RA vs. WT OIR	<0.0001
Ripk3$^{-/-}$ RA vs. Ripk3$^{-/-}$ OIR	<0.0001
WT OIR vs. Ripk3$^{-/-}$ OIR	0.2969

Statistical analysis for P25 samples

Tukey's multiple comparisons test	P-value
WT RA vs. Ripk3$^{-/-}$ RA	0.9975

WT RA vs. WT OIR	0.011
WT RA vs. Ripk3$^{-/-}$ OIR	<0.0001
Ripk3$^{-/-}$ RA vs. WT OIR	0.0167
Ripk3$^{-/-}$ RA vs. Ripk3$^{-/-}$ OIR	<0.0001
WT OIR vs. Ripk3$^{-/-}$ OIR	0.0258

4. Casp8 qPCR

For P7 samples, parametric and unpaired t-test was used

For P12, P17, and P25 samples, One-way ANOVA with Tukey's multiple comparisons test was used

	P7				P12			
	RA		OIR		RA		OIR	
	WT	Ripk3	WT	Ripk3	WT	Ripk3	WT	Ripk3
Mean	0.000742	0.000798	-	-	0.001089	0.001043	0.000800	0.000840
Stdev	0.000185	0.000064	-	-	0.000099	0.000183	0.000056	0.000059

	P17				P25			
	RA		OIR		RA		OIR	
	WT	Ripk3	WT	Ripk3	WT	Ripk3	WT	Ripk3
Mean	0.000904	0.000858	0.001660	0.002439	0.000511	0.000670	0.000849	0.000964
Stdev	0.000109	0.000040	0.000250	0.000199	0.000056	0.000088	0.000151	0.000118

Statistical analysis for P7 samples

t-test	WT	Ripk3$^{-/-}$
P-value		0.5051

Statistical analysis for P12 samples

Tukey's multiple comparisons test	P-value
WT RA vs. Ripk3$^{-/-}$ RA	0.8905
WT RA vs. WT OIR	0.0012
WT RA vs. Ripk3$^{-/-}$ OIR	0.005
Ripk3$^{-/-}$ RA vs. WT OIR	0.0063
Ripk3$^{-/-}$ RA vs. Ripk3$^{-/-}$ OIR	0.0243
WT OIR vs. Ripk3$^{-/-}$ OIR	0.9267

Statistical analysis for P17 samples

Tukey's multiple comparisons test	P-value
WT RA vs. $Ripk3^{-/-}$ RA	0.9639
WT RA vs. WT OIR	<0.0001
WT RA vs. $Ripk3^{-/-}$ OIR	<0.0001
$Ripk3^{-/-}$ RA vs. WT OIR	<0.0001
$Ripk3^{-/-}$ RA vs. $Ripk3^{-/-}$ OIR	<0.0001
WT OIR vs. $Ripk3^{-/-}$ OIR	<0.0001

Statistical analysis for P25 samples

Tukey's multiple comparisons test	P-value
WT RA vs. $Ripk3^{-/-}$ RA	0.0862
WT RA vs. WT OIR	0.0002
WT RA vs. $Ripk3^{-/-}$ OIR	<0.0001
$Ripk3^{-/-}$ RA vs. WT OIR	0.0449
$Ripk3^{-/-}$ RA vs. $Ripk3^{-/-}$ OIR	0.0008
WT OIR vs. $Ripk3^{-/-}$ OIR	0.2879

Figure 7. Increased ICAM-2, F4/80 and collagen I staining following laser photocoagulation in Ripk3$^{-/-}$ mice.

Parametric and unpaired t-test was used

ICAM-2 staining

	WT	Ripk3$^{-/-}$
Mean	11222	19426
Standard deviation	969.1	1934
P-value		0.0002

F4/80 staining

	WT	Ripk3$^{-/-}$
Mean	48388	64617
Standard deviation	4907	4001
P-value		0.0126

Collagen I staining

	WT	Ripk3$^{-/-}$
Mean	21543	34361
Standard deviation	2184	2731
P-value		0.0005

Figure 8. Inhibition of RIPK1 and RIPk3 activity mitigates choroidal neovascularization.

Parametric and unpaired t-test was used

ICAM-2 staining

	Vehicle	C9
Mean	19382	11271
Standard deviation	1644	1103
P-value		<0.0001
